# A genome-wide screen for acrophobia susceptibility loci in a Finnish isolate

**DOI:** 10.1038/srep39345

**Published:** 2016-12-20

**Authors:** Zuzanna Misiewicz, Tero Hiekkalinna, Tiina Paunio, Teppo Varilo, Joseph D. Terwilliger, Timo Partonen, Iiris Hovatta

**Affiliations:** 1 Department of Biosciences, University of Helsinki, Helsinki, Finland; 2 Department of Health, National Institute for Health and Welfare, Helsinki, Finland; 3 Institute for Molecular Medicine Finland, University of Helsinki, Helsinki, Finland; 4 Department of Psychiatry, University of Helsinki and Helsinki University Hospital, Helsinki, Finland; 5 Development of Work and Work Organizations, Finnish Institute of Occupational Health, Helsinki, Finland; 6 Department of Medical Genetics, University of Helsinki, Helsinki, Finland; 7 Department of Psychiatry, Department of Genetics and Development, and Gertrude H. Sergievsky Center, Columbia University, New York NY, USA; 8 Division of Medical Genetics, New York State Psychiatric Institute, New York NY, USA

## Abstract

Acrophobia, an abnormal fear of heights, is a specific phobia characterized as apprehension cued by the occurrence or anticipation of elevated spaces. It is considered a complex trait with onset influenced by both genetic and environmental factors. Identification of genetic risk variants would provide novel insight into the genetic basis of the fear of heights phenotype and contribute to the molecular-level understanding of its aetiology. Genetic isolates may facilitate identification of susceptibility alleles due to reduced genetic heterogeneity. We took advantage of an internal genetic isolate in Finland in which a distinct acrophobia phenotype appears to be segregating in pedigrees originally ascertained for schizophrenia. We conducted parametric, nonparametric, joint linkage and linkage disequilibrium analyses using a microsatellite marker panel, genotyped in families to search for chromosomal regions correlated with acrophobia. Our results implicated a few regions with suggestive evidence for linkage on chromosomes 4q28 (LOD = 2.17), 8q24 (LOD = 2.09) and 13q21-q22 (LOD = 2.22). We observed no risk haplotypes shared between different families. These results suggest that genetic predisposition to acrophobia in this genetic isolate is unlikely to be mediated by a small number of shared high-risk alleles, but rather has a complex genetic architecture.

Acrophobia is a pervasive mental disorder, also known as an irrational fear of heights, affecting approximately five percent of the world’s population^1^. It is a disproportional reaction to a common, rational fear, and can be characterized as apprehension, triggered by elevated spaces or anticipation of them. Acrophobia is classified as a specific phobia under the Diagnostic and Statistical Manual of Mental Disorders (DSM-V), and its aetiology is influenced by both genetic and environmental factors^2^,^3^. While demonstrating high comorbidity rates with various psychiatric disorders and diseases, such as different anxiety disorders or major depression^4^,^5^, acquisition of acrophobia is believed to differ from other phobias. It may be mediated through a non-associative pathway^6^, rather than conditioning or learning from negative or traumatic experiences^7^.

The symptoms of individuals suffering from acrophobia involve changes in behavioural, cognitive and physiological functioning, such as confusion and dizziness, when exposed to heights^7^. Physiologically associated with anomalies in balance control and avoidance behaviour, acrophobia is a consequence of an underlying biological anomaly involving impaired visual detection of body sway^8^. In healthy subjects, posture control is obtained by integrated processing of vestibular, visual and proprioceptive inputs^9^. However, in people suffering from acrophobia, dysfunction in one of the feedback mechanisms may lead to increased dependence on other stimuli. In particular, the presence of vestibular dysfunction causes increased dependence on visual or proprioceptive information to keep balance and constant anticipation of matching the natural oscillation of body sway with visual flow stimulation^7^. This matching is the root cause of the feeling of lack of stability, especially when exposed to high places^10^.

Although the biological mechanisms of acrophobia have been thoroughly investigated^8^, little is known about its molecular basis. As in the case of other complex psychiatric diseases, efforts to localize genetic variants predisposing to acrophobia are hindered by the complexity of the clinical phenotype and the heterogeneity of the studied samples^11^. Therefore, isolated populations offer several advantages in studying the genetic architecture of such complex human diseases, both when rare^12^,^13^ or common^14^ variants are thought to be involved in the genetic aetiology of the condition. Firstly, the enrichment of the risk alleles in an isolated population might help enable their identification. Furthermore, the shared environmental and cultural homogeneity is highly beneficial as potent exposures to environmental risks during the lifetime, possibly influencing the aetiology of the disorder, are not routinely evaluated and might therefore be difficult to detect^11^.

To reduce genetic heterogeneity we have used an isolated homogenous population from north-eastern Finland with small number of founders. This population, described elsewhere^15^,^16^, has been intensively characterized using multiple high-quality historical and health-care registries and was previously studied due to increased lifetime risk of schizophrenia, compared with the rest of Finland^17^. While the collection of the schizophrenia sample was ongoing, we observed that a large number of family members had distinct and severe acrophobia that segregated independently from schizophrenia. In this study, we aimed to map genetic loci predisposing to this specific phenotype. We hypothesized that predisposition to acrophobia may be mediated by a small number of high risk susceptibility variants segregating in this isolate, and that we could possibly identify them through a genome-wide linkage scan using microsatellite markers, which had been genotyped as a part of earlier linkage-based gene mapping studies^17^,^18^.

## Results

**Figure 1:**
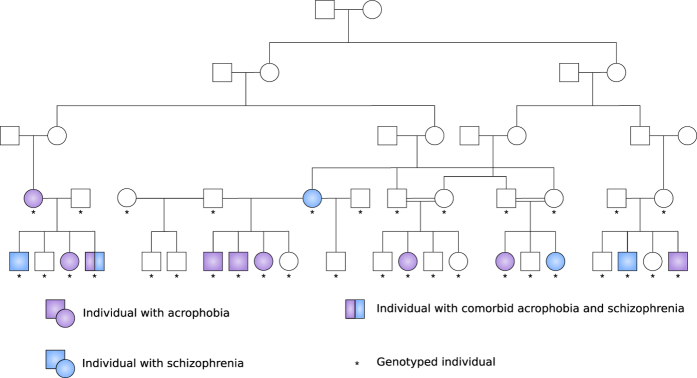
Pedigree structure of the most extended acrophobia pedigree with the largest number of affected individuals.

**Figure 2:**
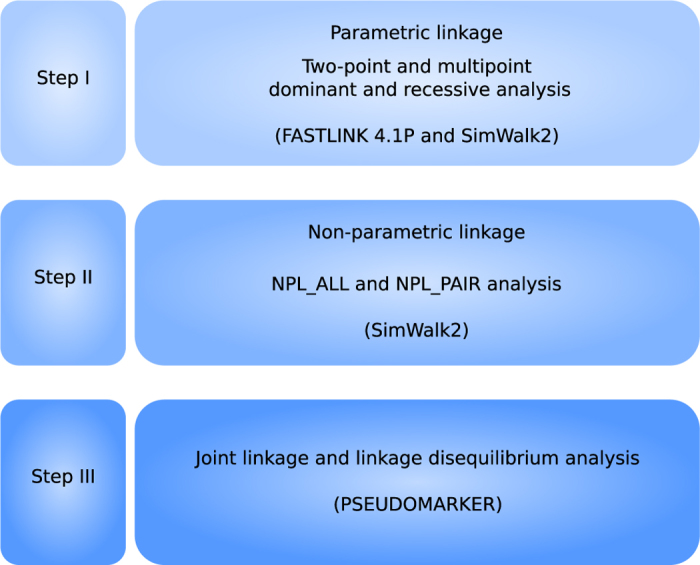
Schematic flowchart of conducted analyses. The name of the software used is given in brackets.

Our sample was composed mostly of large multigenerational pedigrees with multiple individuals affected with acrophobia and at least one parent born in the isolate (Fig. 1). It included 642 people, 42 of them affected only with acrophobia (6.5%) and 75 with acrophobia comorbid with schizophrenia (11.7%; Table 1). As part of earlier studies^17^,^18^, we had genotype information available from 367 individuals, assessed by a genome-wide microsatellite marker panel. As parametric and nonparametric (model-free) methods have different strengths and weaknesses in detecting linkage^19^, we applied both techniques across the genome. Two-point parametric analysis has been proven to be more powerful than nonparametric analysis, provided that the trait and marker locus are specified correctly. However, in case the parametric model is incorrect, the power of the parametric linkage might be exceeded by nonparametric methods. As the inheritance pattern in our study is unknown, we decided to conduct analyses with both methods. In the parametric analysis, we used dominant and recessive models due to unknown inheritance pattern, followed by nonparametric analysis, and joint linkage and linkage disequilibrium analysis (Fig. 2). While two-point analysis examines linkage of a disease to a single marker locus at a time, multipoint analysis evaluates linkage to multiple markers simultaneously. This results in a greater power to detect linkage, but also an inflation of type I error (false positive) if marker-related information is incorrect^20^. We performed multipoint, but not two-point nonparametric analysis, because of the limitations in the size of the pedigrees, which can be analysed, without being divided, by currently available software^21^. Due to the significant number of individuals with acrophobia comorbid with schizophrenia, we analysed all individuals with acrophobia, but also conducted separate analyses with individuals with pure acrophobia and pure schizophrenia to investigate whether any signal was mainly coming from genetic factors influencing susceptibility to schizophrenia independent of acrophobia.

### Parametric two-point recessive and dominant linkage analysis

We first carried out parametric two-point linkage analysis to identify genetic regions linked with acrophobia. The strongest evidence for linkage in the acrophobia sample (including cases with comorbid schizophrenia) was obtained with marker D5S2115 (LOD = 2.16) using a dominant model. In the pure acrophobia sample, the same marker yielded a LOD score of 0.58 (dominant model), suggesting that this finding may be mainly driven by the schizophrenia phenotype. This was further confirmed by analysing all individuals with schizophrenia without comorbid acrophobia (LOD = 1.57, dominant model). For the pure acrophobia sample, we obtained the highest LOD score with marker D8S373 (LOD = 2.09) adopting a recessive inheritance model. Since in the acrophobia with comorbid schizophrenia sample the same marker yielded a LOD score of 0.51 (recessive model) and in the pure schizophrenia sample a LOD score of 0.00 (recessive model), this signal may be produced mainly by the acrophobia phenotype. Table 2 shows the results of the two-point analyses for markers giving the strongest evidence of linkage (LOD score of >1.6).

### Parametric multipoint linkage analysis

We carried out parametric multipoint analysis for chromosomes with markers and models which yielded LOD score of >2.0 in at least one parametric two-point analysis (chromosome 5: dominant model in acrophobia with comorbid schizophrenia; and chromosome 8: recessive model in pure acrophobia). In the acrophobia sample (including cases with comorbid schizophrenia), marker D5S2115 yielded a maximum LOD score of 0.054 (α = 0.15, dominant model). In the pure acrophobia sample the highest LOD score on chromosome 8 was with marker D8S373 (0.533, α = 1.00, recessive model).

### Nonparametric multipoint linkage analysis

**Figure 3:**
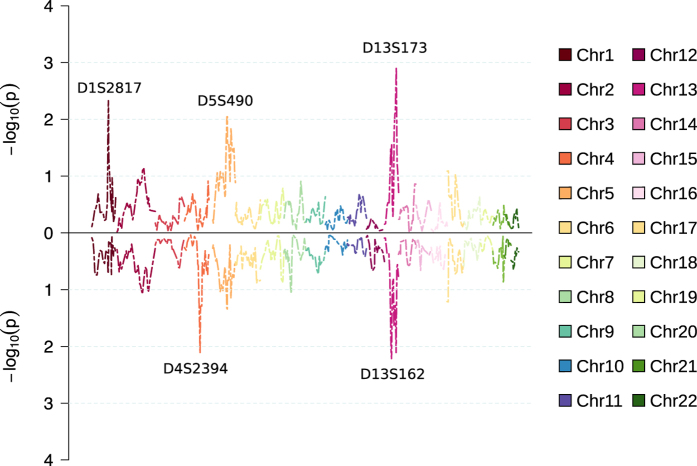
Results from multipoint nonparametric analysis with NPL_ALL model (SimWalk2). The upper panel shows comorbid acrophobia and schizophrenia sample, while the lower panel shows pure acrophobia sample.

We next carried out multipoint nonparametric genome-wide linkage analysis with empirical NPL_ALL model (Fig. 3), measuring if a few founder-alleles are overly represented in affected individuals. Among the acrophobia sample (including cases with comorbid schizophrenia), the maximum LOD score of 2.91 was detected with marker D13S173, while for the pure acrophobia sample the same marker reached LOD score of 2.11. In the pure acrophobia sample the most significant evidence of linkage (LOD score = 2.22) was observed with marker D13S162. Markers D13S173 and D13S162 are located 37.8 cM apart and are therefore not strongly linked.

**Figure 4:**
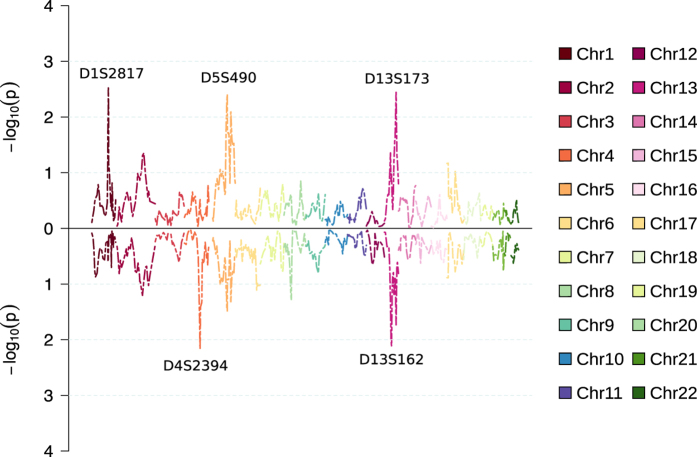
Results from multipoint nonparametric analysis with NPL_PAIR model (SimWalk2). The upper panel shows comorbid acrophobia and schizophrenia sample, while the lower panel shows pure acrophobia sample.

Figure 4 shows the results from the multipoint nonparametric linkage analysis with empirical NPL_PAIR model measuring the sum of conditional kinship coefficients for all affected pairs. For acrophobia sample (including cases with comorbid schizophrenia), NPL_PAIR approach yielded the maximum LOD score with marker D1S2817 (LOD = 2.52), while in the pure acrophobia sample the LOD score for the same marker was 0.47. The strongest finding for NPL_PAIR analysis in the pure acrophobia sample was a LOD score of 2.17 with marker D4S2394.

### Joint linkage and linkage disequilibrium analysis

**Figure 5:**
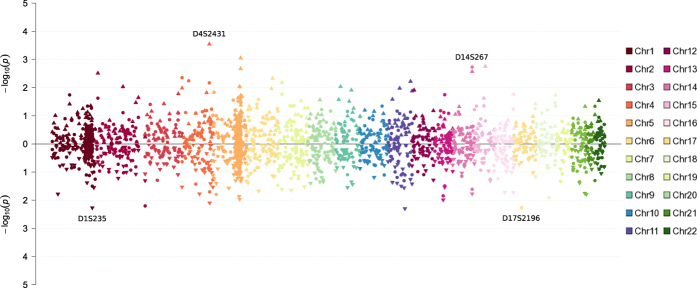
Results from linkage disequilibrium conditional on linkage analysis (PSEUDOMARKER). Recessive analysis is shown with triangles and dominant analysis with dots. The upper panel shows comorbid acrophobia and schizophrenia sample, while the lower panel shows pure acrophobia sample.

We hypothesized that reduced genetic heterogeneity in the genetic isolate would lead to the majority of the cases carrying the same predisposing variant, detectable as linkage disequilibrium (LD). Therefore, we performed PSEUDOMARKER analysis of LD conditional on linkage (Fig. 5). The strongest evidence for association to acrophobia (including cases with comorbid schizophrenia) was detected for markers D4S2431 (P = 0.0003, recessive model) and D14S267 (P = 0.0019, dominant model). In the pure acrophobia sample the same markers yielded P values of 0.1704 and 0.0244, respectively. For the pure acrophobia sample the strongest association was obtained to markers D17S2196 (P = 0.0054, dominant model) and D1S235 (P = 0.0054, recessive model). These results are consistent with the lack of evidence of linkage in the sample.

### Power simulation

To test the statistical power of the analysed sample, we performed simulation with SLINK^22^ software. We obtained average maximum LOD scores of 5.77 and 5.03 with the dominant and recessive model, respectively. Furthermore, 31% of replicates under the dominant and 12% of replicates under the recessive model reached the conventional LOD score threshold of 3.0. Therefore, this sample has adequate statistical power to obtain significant evidence for linkage to major loci predisposing to acrophobia.

## Discussion

We aimed to find genetic loci predisposing to acrophobia in a genetically isolated population with the hypothesis that due to reduced genetic heterogeneity, a few risk alleles predisposing to the phenotype might be identified. While several loci attained LOD score of >2.0, we observed no genome-wide significant evidence for linkage at any of the studied markers.

We detected the strongest evidence of linkage to acrophobia on chromosomal region 13q21-q22 with a peak on marker D13S162 (LOD = 2.22; pure acrophobia sample). To our knowledge, this region has not been previously associated with phobias or other anxiety disorders. SNP rs2323266, located 14.01 Mb from marker D13S162 and close to the protocadherin 20 (*PCDH20*) gene in this region, has been previously connected to positive symptom dimension in a genome-wide association study (GWAS) of schizophrenia (P = 3 × 10^−6^)^23^. However, markers D13S162 and rs2323266 are not located within the same LD block. Thus, in our sample, the finding in this chromosomal region seemed to be specific for acrophobia.

We obtained the second strongest evidence of linkage for acrophobia on chromosomal region 4q28 for marker D4S2394 (LOD = 2.17; pure acrophobia sample). Again, in our study, this signal appeared to be mainly coming from acrophobia and not schizophrenia phenotype (LOD = 0.52; pure schizophrenia sample). This region has not been previously associated with anxiety disorders or schizophrenia.

Chromosomal region 8q24.2-q24.3 with marker D8S373 (LOD = 2.09) was the third region most strongly linked to acrophobia. It has previously been associated with bipolar disorder^24^,^25^,^26^ and schizophrenia^27^. However, in our samples of acrophobia with comorbid schizophrenia (LOD = 0.51) and pure schizophrenia (LOD = 0.00) this region did not provide evidence for linkage. This region encompasses 49 genes, including several candidate genes for psychiatric disorders: potassium voltage-gated channel subfamily KQT member 3 (*KCNQ3*)^24^,^28^ coding for a voltage-gated potassium channel; adenylate cyclase 8 (*ADCY8*)^24^ involved in fear learning and memory, and long-term memory consolidation^29^; Ly6/Neurotoxin 1 (*LYNX1*)^30^ (located on 8q24.3 near D8S373) involved in the development of visual perception including binocular vision and motor learning during the critical period^31^,^32^,^33^,^34^; and activity-regulated cytoskeleton-associated protein (*ARC*)^29^ connected to long-term potentiation and the consolidation of long-term memory. Our strongest evidence for LD conditional on linkage (P = 0.0054, dominant model) was observed with markers located in chromosomal region 17p11. The region harbours hundreds of genes, however, to our knowledge, none of them have been previously directly connected to phobias or other anxiety disorders.

Several of the chromosomal regions we identified seemed mainly specific for schizophrenia. The most significant of those regions were 5q31 and 1q32, discussed above for markers D5S2115 and D1S2817. Both of them have been previously associated with schizophrenia in numerous studies^35^,^36^,^37^,^38^.

Furthermore, several markers on the long arm of chromosome 13, localized between 13q31 and 13q33, showed evidence of linkage (LOD > 2.0) to both pure acrophobia and acrophobia with comorbid schizophrenia, and also weak linkage to pure schizophrenia (maximum LOD = 1.26). Therefore, this region may harbour variants influencing susceptibility to both phenotypes. As specific phobia subtypes have high comorbidity with other anxiety disorders^4^ and psychiatric disorders, such as schizophrenia^39^, the same genetic variants affecting brain function may be involved in both diseases partially explaining the high comorbidity rates and overlapping biological symptoms. Intriguingly, several shared symptoms related to balance control and avoidance behaviour, central in acrophobia, have been described in schizophrenia and depressive disorders. Most importantly, irregularities in eye movement and head coordination propensities associated with difficulties in classification of relevant visual stimuli have been observed in schizophrenia patients^40^. Furthermore, the primary vestibular disfunction leading to chronic dizziness occurs both in specific phobias and depressive disorders^41^. Lastly, acrophobia is a significant predictor of later depressive episodes, generalized anxiety and panic attacks^42^. Together, these observations may be related to partially shared genetic predisposition to acrophobia and other comorbid psychiatric diseases, including schizophrenia.

We recognise the hypothesis-generating character of our study. It included a large number of genotyped markers (570) and analysed models (6). Although this serves to strengthen the study, it inevitably lead to multiple statistical testing. However, as the tests carried out are not completely independent, the adjustment for multiple testing is not straightforward^43^. Consequently, avoidance of the type I error might unintentionally inflate the type II error (false negative). Therefore, we decided to provide the LOD scores and their corresponding uncorrected nominal P values, when applicable, and rely on the future studies of acrophobia in independent samples to assess whether the chromosomal regions identified in this study harbor true predisposing variants.

In recent years, single nucleotide polymorphisms (SNPs) have replaced microsatellite markers due to their lower genotyping costs. This technical advance has enabled genome-wide association studies (GWASs) in which thousands of individuals are genotyped. Consequently, the usage of microsatellite markers, a class of short tandem repeats (STRs), has severely decreased. However, due to their highly polymorphic nature microsatellites are still considered more informative than the diallelic SNPs^44^ in studying the genetic architecture of complex human diseases. Unlike in the population-based association studies, the enrichment of the risk alleles in isolated populations and higher degree of LD may enable the identification of genetic risk factors in a smaller sample and with smaller number of markers, as shown previously for neuropsychiatric diseases, such as schizophrenia and autism^11^,^17^,^45^.

In conclusion, our findings suggest that the genetic basis of acrophobia is highly complex, even in this genetic isolate, as we were not able to identify high-risk variants shared by several families. However, we identified several chromosomal regions with suggestive evidence for linkage which could be investigated further in other acrophobia samples and meta-analyses of such datasets having an increased statistical power.

## Methods

### Study sample

The sample was composed mostly of large multigenerational pedigrees with multiple affected individuals and at least one parent born in the isolate (Fig. 1). It comprised 57 families including 642 people, 75 of them affected with acrophobia and schizophrenia (11.7%) and 42 with pure acrophobia (6.5%). All pedigrees are part of a Finnish severe mental disorders family collection of the National Institute for Health and Welfare^17^,^18^,^46^,^47^. We traced ancestors from Finnish Population Registries and performed a genealogical study in accordance with published criteria^47^.

Data from psychiatric case notes and treatment facilities concerning affected individuals and blood samples were collected between 1991 and 2002^48^. Structured Clinical Interviews for DSM-IV (SCID-I)^49^ followed by a diagnostic assessment performed in accordance with the Diagnostic and Statistical Manual of Mental Disorders, 4^th^ edition (DSM-IV) criteria^50^ and Operational Criteria Checklist (OPCRIT)^44^ were conducted at a later time, between 1998 and 2002^48^. Following the interviews, assessment was undertaken independently by two psychiatrists (T.Par. and R.A.). In case of disagreement, third psychiatrist (J.L.), reanalysed the case in order to gain consensus^17^. The sample used in the study is detailed in Table 1. The ethical review board of the National Institute for Health and Welfare (THL), formerly the National Public Health Institute of Finland (KTL), approved all experimental protocols in this study and the methods used were carried out in accordance with the approved guidelines. Informed consent was obtained from all participants before enrolment in the study.

### Genotyping

We analysed 575 autosomal microsatellite markers across the genome that had been genotyped as a part of earlier linkage-based gene mapping studies^17^,^48^. We had genotype information available from all affected and 292 unaffected individuals.

### Statistical analyses

We performed statistical analyses separately for acrophobia with comorbid schizophrenia and pure acrophobia sample to discriminate between the possible shared and separate genetic signals associated with the phenotypes. We further followed with statistical analysis of the pure schizophrenia sample for the most interesting results. We first checked all genotypes for Mendelian inconsistencies with PedCheck software^51^ and removed erroneous genotypes, which accounted for less than 0.08% of all genotypes. We performed all analyses as affected-only, meaning that all phenotypes of healthy individuals and those with unknown affection status were treated as *unknown*, due to the fact that unaffected family members were not systematically assessed^52^.

### Parametric two-point recessive and dominant linkage analysis

We performed two-point parametric linkage analysis with statistical software package FASTLINK 4.1 P under a recessive and dominant mode of inheritance with, respectively, penetrance of 0.001% and 90%, disease allele frequency of 0.00001 and 0.01, and phenocopy rates of 0 and 0.01. FASTLINK 4.1 P program was implemented in a helper program AUTOGSCAN^53^.

### Parametric and nonparametric multipoint linkage analysis

We carried out multipoint parametric and nonparametric linkage analysis with SimWalk2 version 2.96^54^,^55^,^56^. For the nonparametric analysis, two statistics were examined: empirical NPL_ALL model, measuring if few founder-alleles are overly represented in affected individuals, and NPL_PAIR, measuring the sum of conditional kinship coefficients for all affected pairs. In all analyses, pedigrees with just one, non-related affected, were not analysed by the program, limiting the total number of pedigrees evaluated to five for pure acrophobia and 15 for acrophobia with comorbid schizophrenia sample. Files for SimWalk2 software were prepared with free data conversion program Mega2^57^.

### Stage III: Joint linkage and linkage disequilibrium (LD) analysis

We performed the linkage disequilibrium conditional on linkage analysis with PSEUDOMARKER software package under default dominant and recessive models^58^,^59^. PSEUDOMARKER program uses a likelihood-based method, which combines pedigrees of heterogeneous relationship structures, singletons and others, into one unified analysis.

### Power simulation

The power of the analysed pedigrees to detect linkage was estimated with SLINK simulation program and the replicates were analysed with ISIM analysis program implemented in SLINK package^23^. The analysis was performed under PSEUDOMARKER recessive and dominant models with the assumption of complete linkage (θ = 0.0) between marker and trait locus, and with penetrance and disease allele frequency identical to those used in the linkage analysis. We assumed the disease prevalence of five percent^1^ for acrophobia. The analysis was performed using 100 replications.

## Additional Information

**How to cite this article**: Misiewicz, Z. *et al*. A genome-wide screen for acrophobia susceptibility loci in a Finnish isolate. *Sci. Rep.* **6**, 39345; doi: 10.1038/srep39345 (2016).

**Publisher's note:** Springer Nature remains neutral with regard to jurisdictional claims in published maps and institutional affiliations.

## Figures and Tables

**Table 1:** Data structure and summary statistics of the analysed pedigrees.

Characteristic		Pure acrophobia^a^	Acrophobia with comorbid schizophrenia^b^
Pedigrees	All	57	57
	With affected individuals	29 (50.9%)	46 (80.7%)
Individuals	Male	306 (47.7%)	306 (47.7%)
	Female	336 (52.3%)	336 (52.3%)
Affected individuals	Male	15 (4.5%)	31 (9.2%)
	Female	27 (8.8%)	44 (14.4%)
Genotyped individuals	Affected	42 (100%)	75 (100%)
	Unaffected	325 (54.2%)	292 (51.5%)
Average pedigree size		11.26 (3 to 54 individuals)	11.26 (3 to 54 individuals)

Pedigree characteristics computed with PedStats^60^.

^a^Study conducted on individuals with pure acrophobia, where all individuals with comorbid schizophrenia were excluded from the analysis.

^b^Study conducted on individuals with acrophobia (N = 75), including individuals with comorbid schizophrenia (N = 33).

**Table 2:** LOD scores from the two-point parametric analysis (FASTLINK) using dominant and recessive models.

Marker	Chromosome	Position (cM)	Pure acrophobia		Acrophobia with comorbid schizophrenia	
			Dominant model	Recessive model	Dominant model	Recessive model
D5S2115	5	147.47	0.58	0.39	**2.16**	1.54
D13S173	13	87.49	1.21	0.99	1.44	**1.89**
D1S1728	1	97.17	1.04	1.20	0.52	**1.62**
D8S373	8	163.74	0.32	**2.09**	0.02	0.51
D21S1441	21	7.8	0.25	**1.61**	0.00	0.47

All markers with LOD score above 1.6 (in bold), in any of the models, are included in the Table.
